# Proteome profiling of cadmium-induced apoptosis by antibody array analyses in human bronchial epithelial cells

**DOI:** 10.18632/oncotarget.6738

**Published:** 2015-12-23

**Authors:** Yan-Ming Xu, Dan-Dan Wu, Wei Zheng, Fei-Yuan Yu, Feng Yang, Yue Yao, Yuan Zhou, Yick-Pang Ching, Andy T. Y. Lau

**Affiliations:** ^1^ Laboratory of Cancer Biology and Epigenetics, Department of Cell Biology and Genetics, Shantou University Medical College, Shantou, Guangdong 515041, P. R. China; ^2^ School of Biomedical Sciences, Li Ka Shing Faculty of Medicine, The University of Hong Kong, Hong Kong, P. R. China

**Keywords:** cadmium, human lung cells, antibody array, post-translational modifications, mitochondrial pathway

## Abstract

Protein array technology is a powerful platform for the simultaneous determination of the expression levels of a number of proteins as well as post-translational modifications such as phosphorylation. Here, we screen and report for the first time, the dominant signaling cascades and apoptotic mediators during the course of cadmium (Cd)-induced cytotoxicity in human bronchial epithelial cells (BEAS-2B) by antibody array analyses. Proteins from control and Cd-treated cells were captured on Proteome Profiler^™^ Arrays for the parallel determination of the relative levels of protein phosphorylation and proteins associated with apoptosis. Our results indicated that the p38 MAPK- and JNK-related signal transduction pathways were dramatically activated by Cd treatment. Cd potently stimulates the phosphorylations of p38α (MAPK14), JNK1/2 (MAPK8/9), and JUN; while the phosphorylations of Akt1, ERK1/2 (MAPK3/1), GSK3β, and mTOR were suppressed. Moreover, there was an induction of proapoptotic protein BAX, release of cytochrome *c* (CYCS) from mitochondria, activation of caspase-3/9 (CASP3/9); as well as decreased expression of cell cycle checkpoint proteins (TP53, p21, and p27) and several inhibitors of apoptosis proteins (IAPs) [including cIAP-1/2 (BIRC2/3), XIAP (BIRC4), and survivin (BIRC5)]. Pretreatment of cells with the thiol antioxidant glutathione or p38 MAPK/JNK inhibitors before Cd treatment effectively abrogated ROS activation of p38 MAPK/JNK pathways and apoptosis-related proteins. Taken together, our results demonstrate that Cd causes oxidative stress-induced apoptosis; and the p38 MAPK/JNK and mitochondrial pathways are more importantly participated for signal transduction and the induction of apoptosis in Cd-exposed human lung cells.

## INTRODUCTION

Toxic metal ions represent health threats to human lives. In particular, cadmium (Cd) is one of the important carcinogenic heavy metals that can persist in contaminated water, food and soil [1]. Cd is also present in cigarette smokes and airborne particles of polluted air [2, 3]. Studies of the last two decades have established that the effects of Cd are multifaceted and the cellular response to Cd is concentration-dependent and cell-type specific [4–6], which can lead to oxidative stress, promotion of cell proliferation, acquisition of apoptotic resistance, as well as malignant transformation [7–15]. Since lung is one of the susceptible organs prone to Cd-induced cytotoxicity and carcinogenicity [2], therefore, it becomes an urgent matter to understand more on the action mechanisms of Cd to the lungs.

In the past, although many studies have been conducted by scientists to examine the cellular response of cells challenged with Cd, yet, a more systematic approach for the determination of changes at post-translational levels (such as phosphorylation) is lacking [16–21]. Proteomics is a powerful tool to study many proteins in a single analysis. Therefore, in this study, we resolved to screen and report for the first time, the dominant signaling cascades and apoptotic mediators during the course of Cd-induced cytotoxicity in normal human bronchial epithelial cells (BEAS-2B) by antibody array analyses. Proteins from control and Cd-treated cells were captured on Proteome Profiler^™^ Arrays for the parallel determination of the relative levels of expression of a panel of phosphorylated proteins and proteins associated with apoptosis.

To this end, we identified the proteome changes associated with Cd induced cytotoxicity in human bronchial epithelial cells, including ROS activation of the p38 MAPK/JNK pathways and induction of proteins involved in intrinsic apoptosis. On the other hand, the key players of survival signaling pathways (Akt1 and ERK1/2) were suppressed and there were decreased expression of cell-cycle checkpoint proteins (TP53, p21 and p27) and several inhibitors of apoptosis proteins (IAPs), including cIAP-1/2, XIAP, and survivin. Pretreatment of cells with the thiol antioxidant glutathione (GSH) or p38 MAPK/JNK inhibitors before Cd treatment effectively abrogated ROS activation of p38 MAPK/JNK pathways and apoptosis-related proteins. The implications of these findings are that Cd causes oxidative stress-induced cytotoxicity in human lung cells primarily through the p38 MAPK/JNK and mitochondrial pathways; and the differentially-expressed protein signatures could be considered for potential use as biomarkers upon Cd exposure.

## RESULTS

### The p38 MAPK- and JNK-related signal transduction pathways are dramatically activated by Cd treatment

**Figure 1:**
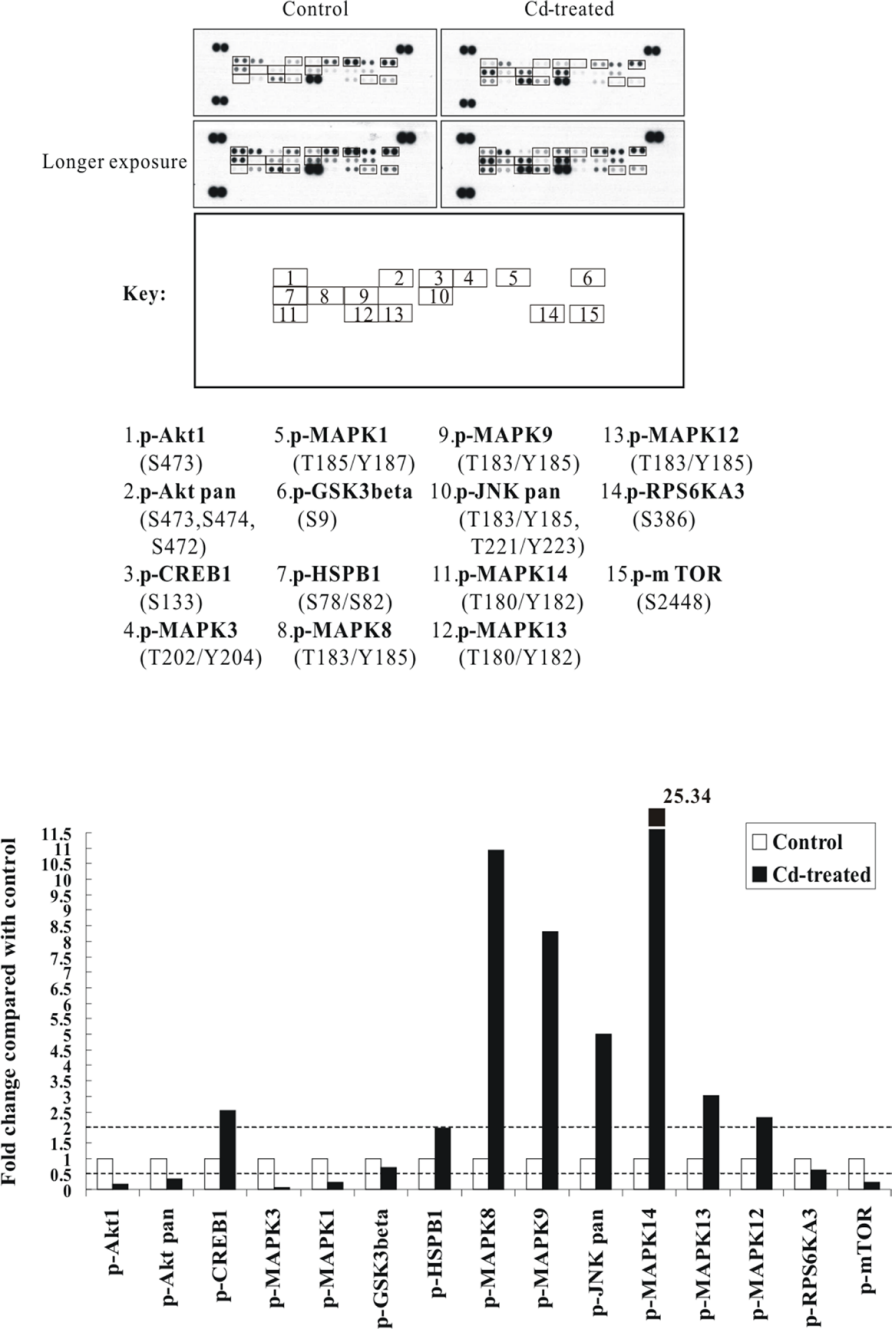
The p38 MAPK/JNK (MAPK14 or MAPK8/9) signaling pathways are strongly activated by Cd treatment in BEAS-2B cells Protein extract (300 μg) were used for phospho-MAPK array analysis. Array spots were visualized in accordance with the manufacturer's instructions. The intensity of each spot was measured as described in “Materials and Methods”. The graph shows the relative fold change of proteins with significant difference upon Cd treatment, setting 1 for control (no treatment of Cd). Protein levels with higher than ± 2 folds (i.e. ≥ 2 or≤ 0.5 as indicated by dotted lines) are considered as candidates that are more importantly participated in the Cd toxicity pathway. The data are shown as an average of two individual sets of sample.

From our previous study, we examined the cytotoxicity of CdCl_2_ in human bronchial epithelial cells (BEAS-2B) and showed that the lethal concentration range is around 20–30 μM post 36 h of CdCl_2_ treatment [22]. In this study, we therefore wish to examine the dominant signaling cascades and apoptotic mediators during the course of Cd-induced cytotoxicity. First, to dissect the early cellular response to Cd, BEAS-2B cells were sham-exposed or treated with 100 μM CdCl_2_ for 3 h (a higher concentration of Cd has been employed as in many studies to study early signaling events) [11, 12, 23]. After treatment, total cell extracts were isolated and subjected to human phospho-MAPK array analysis. Overall, the results indicated that the p38α (MAPK14) and JNK1/2 (MAPK8/9) showed the greatest fold of increase in phosphorylations (with MAPK14 increased 25.34-fold, MAPK8 10.93-fold, and MAPK9 8.31-fold), as compared with the untreated control (Figure 1). Other proteins which also showed increase of phosphorylations included CREB1 (2.54-fold) and HSP27 (HSPB1) (1.98-fold). On the other hand, the phosphorylations of Akt1, mTOR, GSK3β, ERK1/2 (MAPK3/1), and RSK2 (RPS6KA3) were all significantly decreased (Figure 1; Supplementary Table S4).

### Cd activates the p38 MAPK- and JNK-related signal transduction pathways in a time-dependent manner

**Figure 2:**
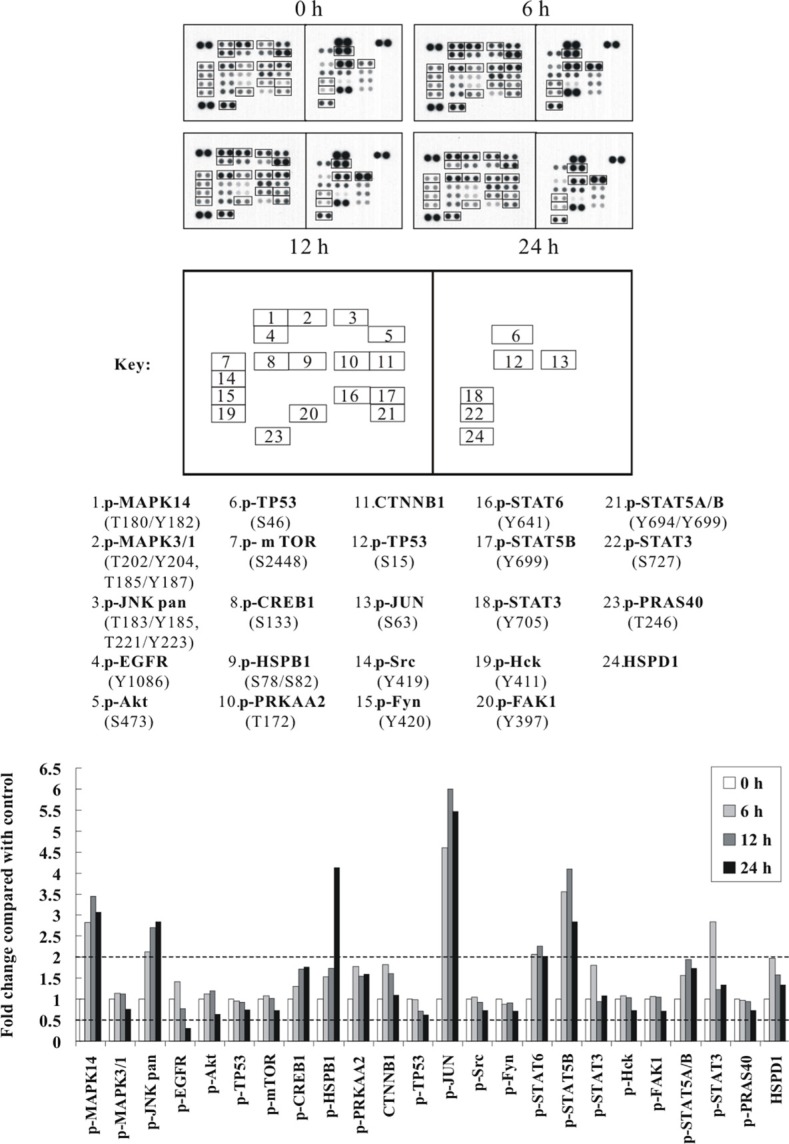
The p38 MAPK/JNK (MAPK14 or MAPK8/9) signaling pathways and c-Jun are strongly activated by Cd treatment in a time-dependent manner Protein extract (600 μg) were used for phospho-kinase array analysis. Array spots were visualized in accordance with the manufacturer's instructions. The intensity of each spot was measured as described in “Materials and Methods”. The graph shows the relative fold change of proteins with significant difference upon Cd treatment, setting 1 for control (no treatment of Cd). Protein levels with higher than ± 2 folds (i.e. ≥ 2 or ≤ 0.5 as indicated by dotted lines) are considered as candidates that are more importantly participated in the Cd toxicity pathway. The data are shown as an average of two individual sets of sample.

To further identify the primary signal transduction pathway induced by Cd, we used a human phospho-kinase array comprising 43 kinase phosphorylation sites, including also kinases from the phospho-MAPK array. This time, BEAS-2B cells were sham-exposed or treated with 30 μM CdCl_2_ for 6, 12, and 24 h. After treatment, total cell extracts were isolated and subjected to human phospho-kinase array analysis. As shown in Figure 2, again, the p38 MAPK- and JNK-related signal transduction pathways were strongly activated by Cd treatment (with MAPK14 increased 3.45-fold and MAPK8/9 2.85-fold). HSPB1, CREB1 and JUN (c-Jun), which are well-known substrates of p38 MAPK and JNK, were also dramatically elevated in phosphorylation upon Cd treatment (with HSPB1 increased 4.14-fold, CREB1 1.76-fold, and JUN 6.00-fold) (Figure 2). Consistent with the phospho-MAPK array results, some of the key players of survival signaling and energy metabolic pathways (p-Akt1, p-mTOR, p-EGFR and p-MAPK3/1), were significantly suppressed (Figure 2; Supplementary Table S5). Interestingly, we found that the phosphorylations of TP53 at S15 and S46 (which are important phosphorylation sites for stress responses) were also decreased, whereas several of the STAT family members, including STAT3, STAT5A/B and STAT6, were all increased. Lastly, the levels of p-PRKAA2 (p-5′-AMP-activated protein kinase catalytic subunit alpha-2), CTNNB1 (Catenin beta-1), and HSPD1 (HSP60) were also moderately increased, while the autophosphorylation levels of several tyrosine kinases like Src, Fyn, Hck, FAK1, as well as the phosphorylation levels of 40 kDa proline-rich Akt substrate (PRAS40), were moderately decreased (Figure 2; Supplementary Table S5).

To ensure if the altered phosphorylation in phospho-MAPK and phospho-kinase arrays are due to upstream regulation instead of simply due to altered protein amounts, we performed western blot analyses using the same cell lysates that were used for antibody array analyses (Supplementary Figure S1A). Our results indicated that among the majority of proteins with increased or decreased in phosphorylations, except for HSPB1 and TP53, most of them have no alteration in total protein levels, indicating that the altered phosphorylation is likely due to upstream regulation.

### The effects of Cd exposure on apoptosis-related proteins in BEAS-2B cells

**Figure 3:**
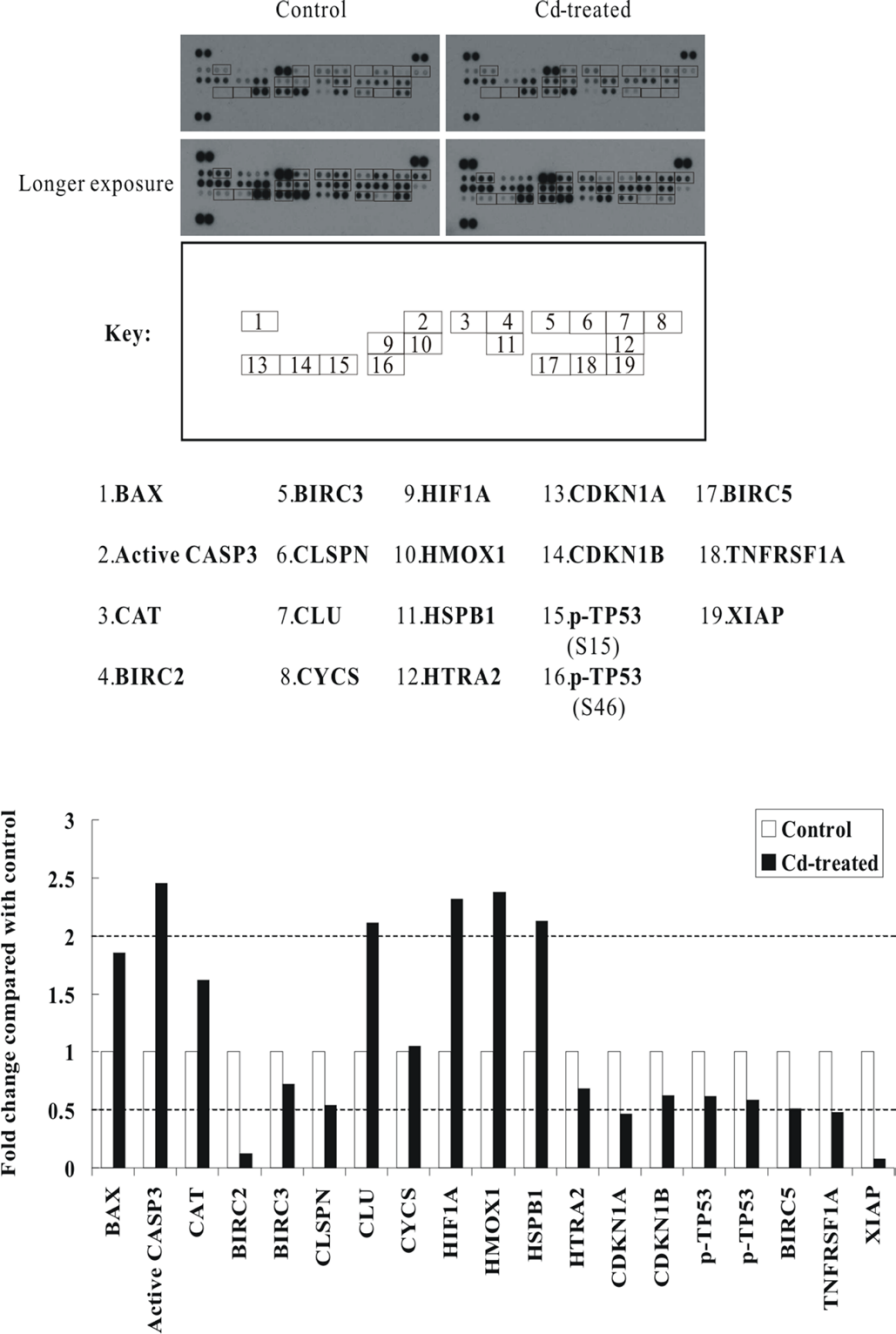
Alterations of apoptosis-related proteins in Cd-treated BEAS-2B cells Protein extract (400 μg) were used for apoptosis array analysis. Array spots were visualized in accordance with the manufacturer's instructions. The intensity of each spot was measured as described in “Materials and Methods”. The graph shows the relative fold change of proteins with significant difference upon Cd treatment, setting 1 for control (no treatment of Cd). Protein levels with higher than ± 2 folds (i.e. ≥ 2 or ≤ 0.5 as indicated by dotted lines) are considered as candidates that are more importantly participated in the Cd toxicity pathway. The data are shown as an average of two individual sets of sample.

Next, we examined the apoptotic mediators that drive cell death in Cd-treated BEAS-2B cells. We employed the human apoptosis array with 35 apoptosis related-proteins. BEAS-2B cells were sham-exposed or treated with 30 μM CdCl_2_ for 24 h, total cell extracts were isolated and then subjected to human apoptosis array analysis. As shown in Figure 3, the proapoptotic protein BAX and active caspase-3 (CASP3) were increased by 1.85-fold and 2.45-fold, respectively. Several other proteins were also induced, like catalase (CAT) (1.62-fold), clusterin (CLU) (2.11-fold), hypoxia-inducible factor 1-alpha (HIF1A) (2.31-fold), and heme oxygenase 1 (HMOX1) (2.38-fold). Interestingly, the expression of several inhibitors of apoptosis proteins (IAPs), including cIAP-1 (BIRC2), cIAP-2 (BIRC3), XIAP (BIRC4), and survivin (BIRC5), were significantly suppressed. Other proteins which showed significant suppression included claspin (CLSPN) [an essential replication checkpoint control protein], high temperature requirement protein A2 (HTRA2) [a serine protease that is involved in the degradation of aberrantly folded proteins during conditions of cellular stress], cyclin-dependent kinase inhibitor 1 (CDKN1A/p21), cyclin-dependent kinase inhibitor 1B (CDKN1B/p27), and tumor necrosis factor receptor superfamily member 1A (TNFRSF1A) (Figure 3; Supplementary Table S6). Again, to confirm the data from the apoptosis array analyses, we performed western blot analyses using the same cell lysates that were used for antibody array analyses (Supplementary Figure S1B). Our results indicated that similar trend of induction or repression can be observed as they appeared in antibody array analyses, suggesting that the data from apoptosis arrays are reliable.

### Induction of proapoptotic protein BAX and the loss of mitochondrial transmembrane potential in Cd-treated BEAS-2B cells

**Figure 4:**
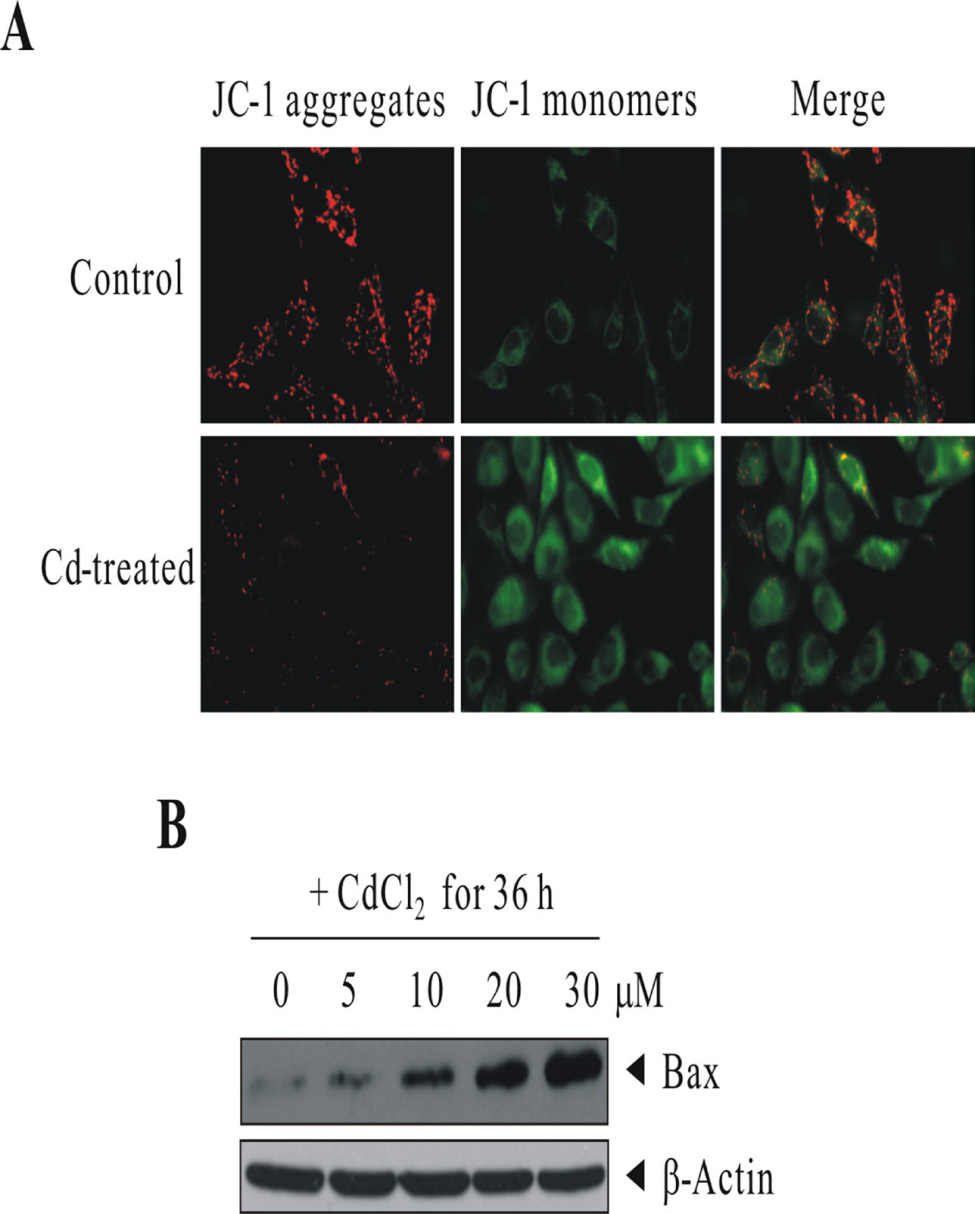
Cd treatment induced the loss of mitochondrial transmembrane potential and the up-regulation of proapoptotic protein BAX (**A**) BEAS-2B cells were sham-exposed or dosed with 30 μM CdCl_2_ for 18 h. JC-1 assay was conducted as described in “Materials and methods” for the determination of mitochondrial membrane integrity. (**B**) BEAS-2B cells were sham-exposed or dosed with increasing concentrations of CdCl_2_ for 36 h; cells were lysed; and protein extracts were subjected to western blot analysis using antibodies against BAX. The same blot was stripped and reprobed with the monoclonal β-actin antibody to monitor the loading difference. The results are representative of three independent experiments.

Since 30 μM CdCl_2_ treatment induced cell death in BEAS-2B cells with the induction of proapoptotic BAX (from human apoptosis array results), we therefore would like to check the integrity of mitochondria. We used the JC-1 Mitochondria Apoptosis Detection Kit which can distinguish healthy and apoptotic cells by detecting the loss of the mitochondrial transmembrane potential (Δψ). BEAS-2B cells were sham-exposed or dosed with 30 μM CdCl_2_ for 18 h. JC-1 assay was conducted as described in Materials and Methods for the determination of mitochondrial membrane integrity. As we can see in Figure 4A, JC-1 aggregates (red colors) were robustly observed in control cells which indicate cells are healthy and with an intact mitochondrial potential. However, for Cd-treated cells, the red colors were visibly-diminished, suggesting the collapses of mitochondrial potential, and JC-1 dye is no longer accumulated within the mitochondria, but dispersed to the cytoplasm (green colors). To further verify the results from apoptosis array that BAX is induced upon Cd treatment, BEAS-2B cells were sham-exposed or dosed with increasing concentrations of CdCl_2_ for 36 h; cells were lysed; and protein extracts were subjected to western blot analysis using antibodies against BAX (Figure 4B). The results indicated that BAX is induced in a dose-dependent manner, which would likely accelerate programmed cell death by binding to mitochondrion membrane, leading to the release of cytochrome *c* (CYCS), promoting activation of caspases, and triggering apoptosis.

### Inhibition of oxidative stress by GSH abrogated the activation of Cd-induced p38/JNK pathways and proteins involved in apoptosis signaling

**Figure 5:**
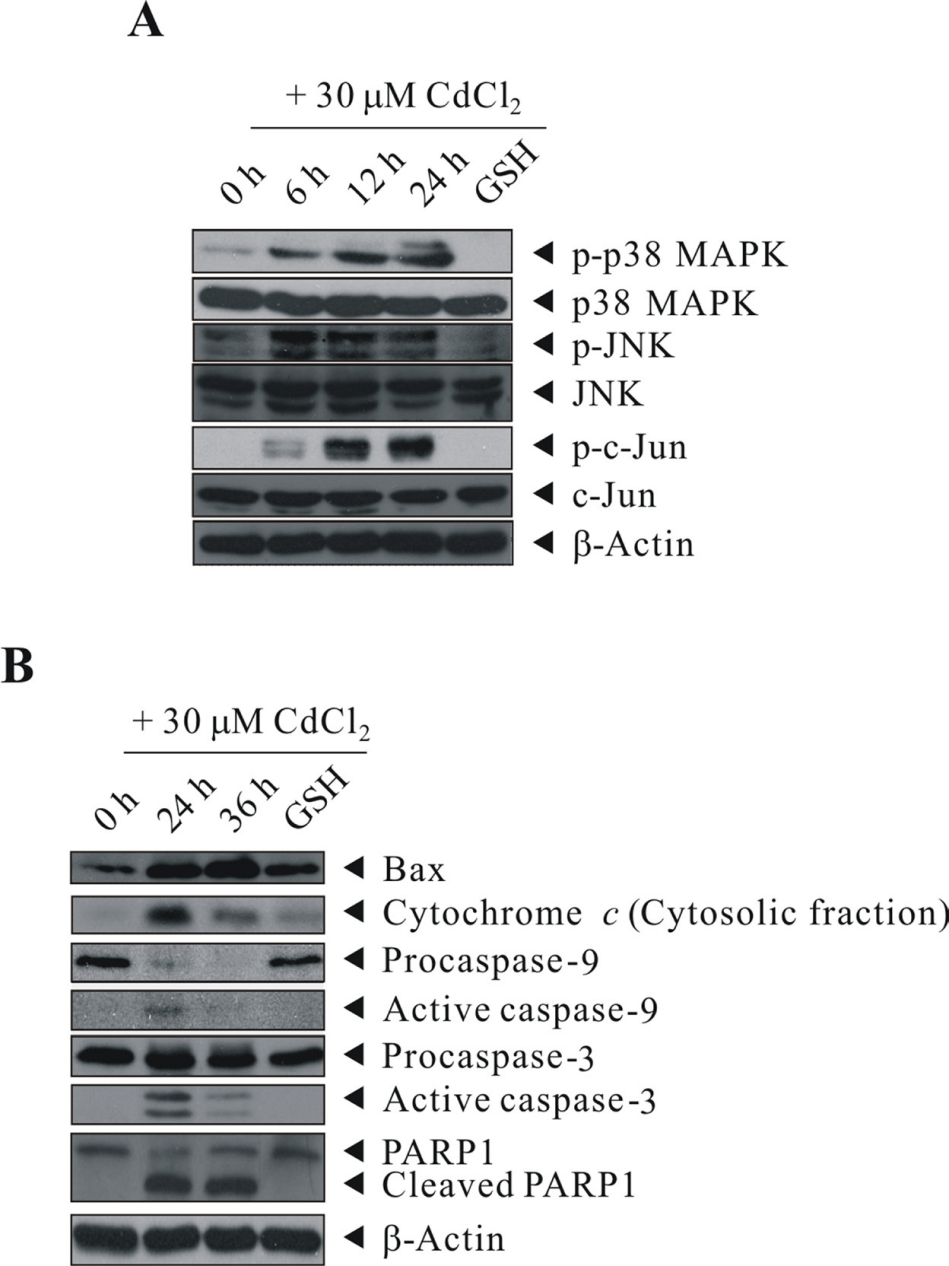
GSH inhibited the activation of Cd-induced p38 MAPK/JNK pathways and proteins involved in apoptosis signaling BEAS-2B cells were sham-exposed or dosed with 30 μM CdCl_2_ for 6, 12, or 24 h (**A**) or with 30 μM CdCl_2_ for 24 or 36 h (**B**); cells were lysed; and protein extracts were subjected to western blot analyses using antibodies against p-p38 MAPK, p38 MAPK, p-JNK, JNK, p-c-Jun, c-Jun, Bax, Cytochrome *c*, Caspase-9, Caspase-3, and PARP1. Cells were also pretreated with 20 mM GSH for 1 h before exposed to 30 μM CdCl_2_ for 24 (A) or 36 h (B). The same blot was stripped and reprobed with the monoclonal β-actin antibody to monitor the loading difference. The results are representative of three independent experiments.

To determine whether the data from kinase array analyses were reliable, we used the same cell lysate for western blot analysis. As shown in Figure 5A, treatment of BEAS-2B cells with 30 μM of CdCl_2_ induced the activation of p38 MAPK, JNK and c-Jun in a time-dependent manner. Moreover, we checked the expression levels of other important apoptosis mediators that covered in the apoptosis array by western blot analysis [we also performed on caspase-9 (CASP9) and poly [ADP-ribose] polymerase 1 (PARP1) as they were not included in the format of protein arrays]. In the previous section, although the results from apoptosis array showed that there is no difference in CYCS levels between control and Cd-treated cells, however, we wonder that possibly because of no prior subcellular fractionation (just a whole cell lysate analysis), which didn't address whether there is release of CYCS from mitochondria to the cytosol. Therefore, we performed subcellular fractionation and examine again the level of CYCS. This time, we can see that indeed CYCS is increased in the cytosolic fraction upon Cd treatment (Figure 5B). The induction/cleavage of BAX/CYCS/CASP9/CASP3/PARP1 can well be observed, suggesting that Cd-induced apoptosis is likely executed through the intrinsic mitochondrial pathway.

Our previous study reported that Cd-induced cytotoxicity through oxidative stress and along with the expression of a panel of stress/defense proteins, and the Cd-induced cytotoxicity can be blocked by pretreating the cells with the thiol antioxidant GSH [22, 24]. Therefore, we investigated the protective ability of intracellular GSH in the induction/repression of some of the important proteins that we identified in this study. First, we further proved that Cd-induced oxidative stress is due to ROS generation, as we can see that treatment with CdCl_2_ resulted in a rapid elevation of ROS generation in BEAS-2B cells as early as 30 min-treatment (Supplementary Figure S2). As shown in Figure 5, pretreatment of cells with GSH efficiently abrogated the activation of p38 MAPK, JNK, and c-Jun (Figure 5A, lane 5), moreover, the same trend also occurred to BAX, CYCS, CASP9, CASP3, and PARP1, in which their induction or cleavage are blocked by GSH pretreatment (Figure 5B, lane 5).

### Inhibition of Cd-induced p38/JNK activity by specific kinase inhibitors, SB203580 and JNK inhibitor VIII

**Figure 6:**
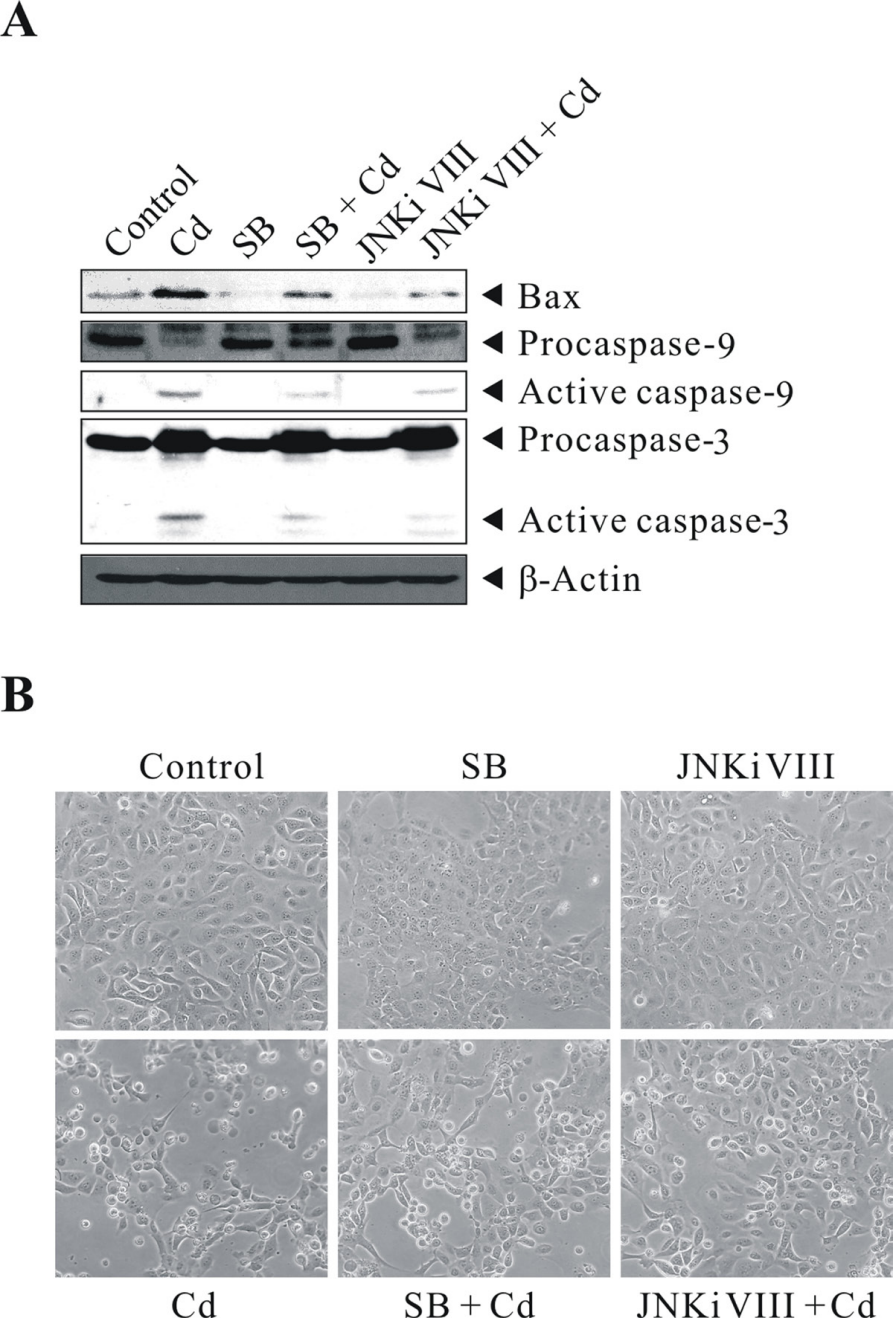
Cd-induced p38/JNK activity correlates with cell death BEAS-2B cells were left untreated or pretreated with 20 μM SB203580 or 40 μM JNK inhibitor VIII for 1 h before exposure to 30 μM CdCl_2_ for 24 h. They were then subjected to (**A**) western blot analysis to detect the levels of Bax, Caspase-9, and Caspase-3. The same blot was stripped and reprobed with the monoclonal β-actin antibody to monitor the loading difference. (**B**) The corresponding cell morphology of BEAS-2B cells under light microscope. The results are representative of three independent experiments; SB, SB203580; JNKi VIII, JNK inhibitor VIII.

To show that a high level of Cd exposure induces the p38/JNK pathways and leads to apoptosis, BEAS-2B cells were left untreated or pretreated with 20 μM SB203580 or 40 μM JNK inhibitor VIII for 1 h before exposure to 30 μM Cd for 24 h. They were then subjected to western blot analysis for the levels of BAX, CASP9, and CASP3. 30 μM Cd treatment stimulated BAX, active CASP9 and CASP3, compared with controls (Figure 6A). This stimulation can be blocked by SB203580/JNK inhibitor VIII (although not as effective as by GSH pretreatment). In the absence of p38/JNK inhibitor pretreatment, massive cytotoxicity can be observed by Cd treatment (Figure 6B). These results suggest that cell death correlates with Cd-induced p38/JNK activity in BEAS-2B cells. Altogether, these data confirm that the p38 MAPK and JNK signaling pathways are critical for mediating the ROS stress signal exerted by Cd. Also, the mitochondrial pathway accelerates programmed cell death, due to the release of CYCS and the subsequent activation of CASP9/3, and the execution of cleavage of a variety of caspase substrates, including PARP1; which are key indicators of intrinsic apoptosis.

## DISCUSSION

In the past, many studies have been conducted to examine the cellular response of cells challenged with toxic metal(s), however, to our knowledge, a more systematic approach for the determination of changes at post-translational levels (such as phosphorylation) is lacking [16–21]. Therefore, it is important to further study the cellular effects at post-translational levels with the aim of identifying proteins whose activities are specifically modified by toxic metal exposure. For instance, most kinase signaling pathways are stimulated rapidly by post-translational modifications, such as phosphorylation of downstream proteins and effectors; while many other proteins are controlled by proteolytic cleavage (such as caspases) and the subcellular localization of proteins are critical determinants of their cellular activities. All these cannot be determined merely at transcriptional levels.

In this study, we resolved to screen and report for the first time, the dominant signaling cascades and apoptotic mediators during the course of Cd-induced cytotoxicity in normal human bronchial epithelial cells (BEAS-2B) by antibody array analyses. Proteins from control and Cd-treated cells were captured on Proteome Profiler^™^ Arrays for the parallel determination of the relative levels of expression of a panel of phosphorylated proteins and proteins associated with apoptosis. This would hopefully provide a better understanding of the mechanisms of action of Cd and allow development of sensitive and specific biomarkers of both exposure and susceptibility for use in both mechanistic and epidemiological studies. However, we want to emphasize for the fact that currently there is no corresponding expression-array available for this commercial phospho-kinase array, therefore, whether the altered phosphorylation is due to upstream regulation or simply due to altered protein amounts is uncertain. To this end, we therefore performed western blot analyses using the same cell lysates that were used for protein array analyses on most of the major proteins that showed significant difference between control and Cd-treatment (Supplementary Figure S1A). Our results indicated that among the majority of proteins with increased or decreased in phosphorylations, except for TP53 and HSPB1, most of them have no alteration in total protein levels, indicating that the altered phosphorylation is likely due to upstream regulation.

Although it is likely that the decreased phosphorylation levels (S15 and S46) of TP53 observed in protein array experiments are merely due to reduced expression of TP53, no matter how, we believe that the total protein amount of TP53 is obviously decreased significantly. The consequence of p53 under-expression is significant as p53 is the principal guardian in maintaining genome stability such that reduced or diminished p53 could then lead to defects in cell cycle check points and DNA repair. As a matter of fact, our western blot analysis also confirmed the decreased expression of p21 and p27 in Cd-treated cells, which may further render the cells deficient in cell cycle arrest for proper repair. For HSPB1, it is well documented that its protein and phosphorylation levels are increased during stress condition; in our cell model, the increased phosphorylation levels (S78 and S82) of HSPB1 observed in phospho-MAPK and phospho-kinase arrays are likely due to up-regulation of HSPB1 protein amount, since we also observed the increase of HSPB1 protein level in apoptosis array and western blot analysis. For HSPD1, it is implicated in mitochondrial protein import and macromolecular assembly, its elevation may prevent misfolding and promote the refolding and proper assembly of unfolded polypeptides generated under stress conditions in the mitochondrial matrix.

**Figure 7:**
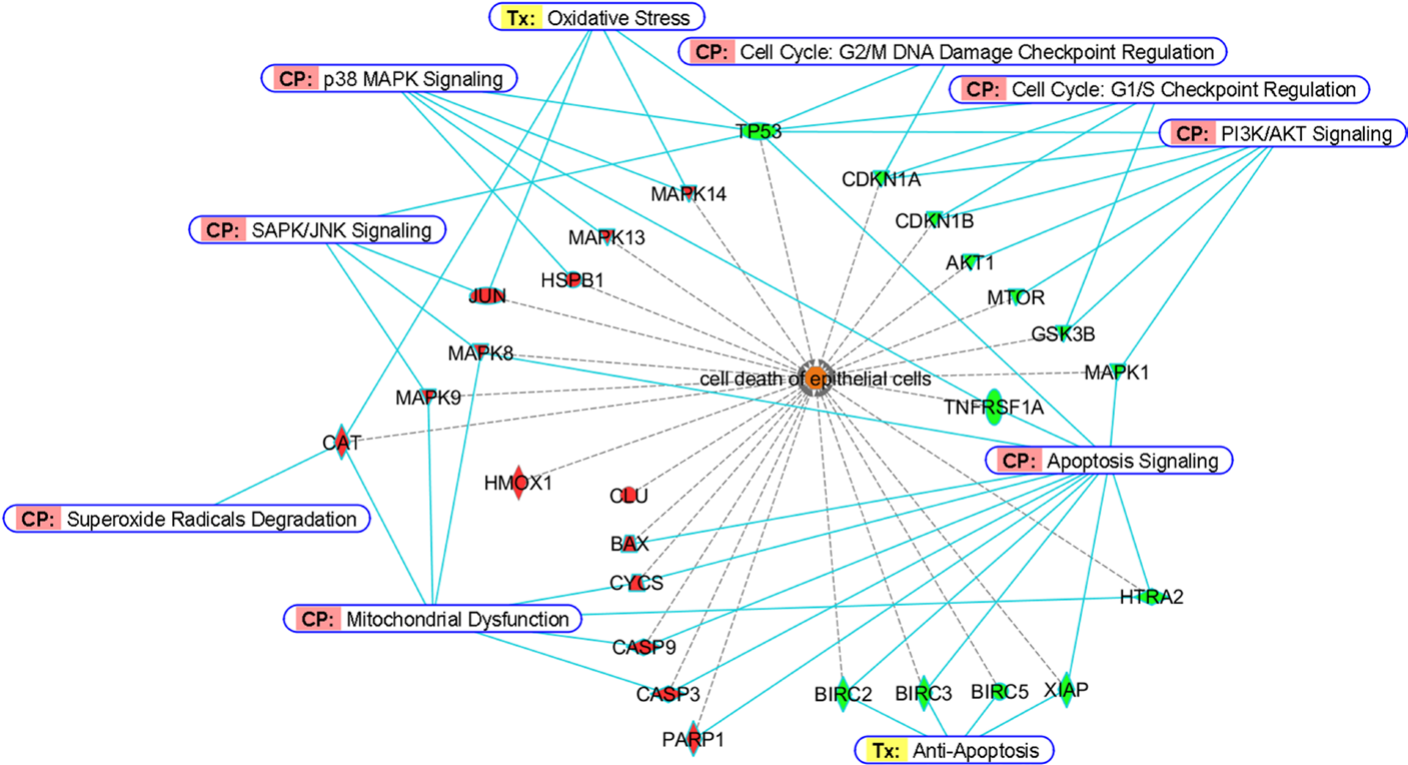
Bioinformatic analyses of differentially-expressed proteins from BEAS-2B cells dosed with Cd Proteins involved in cell death of epithelial cells were classified by Diseases and Bio Functions Annotation from Ingenuity^®^ Pathway Analysis. Red and green colors represent stimulated or inhibited protein expressions/activities, respectively. For CASP9, CASP3 and PARP1, red colors indicate an increase of their cleavage products. Canonical Pathway (CP) and Ingenuity^®^ Tox List (Tx) were also annotated (solid blue lines) to indicate the specific pathway/related biological process among these proteins.

By analyzing the protein list in IPA^®^ database, we are able to examine their relationships in details and summarize the more importantly participated signaling molecules and apoptotic mediators in this study. For a better visualization of the results, we basically clustered the stimulated/activated proteins (red colors) on the left half while inhibited/suppressed proteins (green colors) on the right half as shown in Figure 7. So, upon Cd insults, oxidative stress arises from ROS generation, which activates the p38 MAPK- and SAPK/JNK- signaling and related downstream cascades and stimulates stress gene expressions. The upregulation of antioxidant enzyme CAT in cells served to remedy the imbalance of redox status by degrading superoxide radicals. HMOX1 was also induced, it has been reported that HMOX1 is highly inducible by its substrate heme and by various non-heme substances such as heavy metals, growing evidence shows that HMOX1 can exert antiproliferative and antiapoptotic effects and participate in general cellular defense mechanism against oxidative stress in mammalian cells [25, 26]. Our data are in agreement with previous reports which demonstrated oxidative stress-induced CAT and HMOX1 up-regulations in human BEAS-2B and rat lung epithelial cells [27, 28]. Meanwhile, the expression of CLU was also significantly elevated in Cd-treated BEAS-2B cells. The function of CLU in promotion of cell death has been demonstrated in cultured mouse seminiferous tubules [29]. However, it has also been shown that mitochondrial CLU isoforms suppress BAX-dependent release of CYCS into the cytoplasm and inhibit apoptosis [30]. Nevertheless, if the intracellular detoxification/anti-apoptosis/survival system cannot cope with the detrimental effects exerted by Cd, apoptotic cascade has to be inevitably activated in order to eliminate damaged cells. Our data showed that the proapoptotic protein BAX is stimulated in BEAS-2B cells dosed with Cd. We also demonstrated that CYCS is release to the cytosol (CYCS release is a key indicator of intrinsic apoptosis). The upregulation of BAX and release of CYCS led to mitochondrial dysfunction (as supported by JC-1 assay on loss of mitochondrial potential); causing gradual failure of energy powerhouse of the cells, and subsequently activated the cleavage of CASP9/3 and PARP1. On the other hand, the expressions of cell cycle checkpoint (G_1_/S or G_2_/M) proteins, like TP53, p21 and p27 are all suppressed, which may likely making cells deficient in cell cycle arrest for proper repair. Also, the suppressed expression of TNFRSF1A may lead to inhibition of cell survival [31]. Moreover, the phosphorylations of several key players in cell survival and energy metabolic pathways (Akt1, mTOR, GSK3β and MAPK1) as well as the expressions of inhibitors of apoptosis proteins (BIRC2/3/4/5) were all diminished, all these events finally contributed to the cell death of BEAS-2B lung cells.

Although the IPA^®^ analysis cannot provide a plausible relationship of various STATs (STAT3/5A/5B/6) and HIF1A to cell death of normal human bronchial epithelial cells that we reported here, we are of the opinion that they may also be important factors that are involved during the course of Cd-induced apoptosis. It has been shown that chronic low concentrations of Cd exposure increases HIF1A and VEGF expression through ROS, ERK, and Akt signaling pathways and induces malignant transformation of human bronchial epithelial cells [32], and Cd induced STAT3 activation in HepG2 cells and STAT1/3 phosphorylations in renal proximal tubular epithelial cells [33, 34]. However, no reports have indicated so far for Cd's effects on the phosphorylations of STAT5A/B and STAT6, which warrant further investigation. Suffice to say, our data showed that the ROS generation and activation of p38 MAPK- and JNK-related signal transduction pathways are vital in mediating Cd-induced apoptosis in normal human lung cells. Since pretreatment of GSH or p38/JNK inhibitors prior to Cd effectively abrogated the activation of p38 MAPK/JNK pathways and apoptosis-related proteins in this cell type. Besides, the emergence of mitochondrial dysfunction, deficiency of cell cycle checkpoint proteins, decreased expression of IAPs and diminished survival signaling are also critical which finally contributed to the death fate of BEAS-2B epithelial cells (Figure 7).

In conclusion, we have presented a more global and updated picture of Cd-induced apoptosis in normal human lung cells. Our study also demonstrated the competency of antibody array technology in toxicological research, which might hopefully identify potential biomarkers and lead to the development of agents that can antagonize the cytotoxic effects exerted by toxicants.

## MATERIALS AND METHODS

### Materials

Cadmium chloride (CdCl_2_) was purchased from Sigma Aldrich (St. Louis, MO). The p38 MAPK inhibitor SB203580 (sc-3533) was purchased from Santa Cruz Biotechnology (Santa Cruz, CA). The JNK inhibitor VIII (# 15946) was purchased from Cayman Chemical (Ann Arbor, MI). The Human Phospho-MAPK, Human Phospho-Kinase, and Human Apoptosis Array Kits were obtained from R & D Systems (Minneapolis, MN). The Subcellular Protein Fractionation Kit for Cultured Cells was from Thermo Scientific (Rockford, IL). All other general chemicals were purchased from GE Healthcare (Uppsala, Sweden) and Sigma Aldrich. Antibodies used for western blot were purchased from Santa Cruz Biotechnology, GeneTex (Irvine, CA), Cell Signaling Technology (Danvers, MA) and Sigma Aldrich, with the following dilutions: p-p38 MAPK (4511; Cell Signaling Technology), 1:1000; p38 MAPK (8690; Cell Signaling Technology), 1:1000; p-JNK (sc-293138; Santa Cruz Biotechnology), 1:1000; JNK (sc-7345; Santa Cruz Biotechnology), 1:1000; p-c-Jun (sc-822; Santa Cruz Biotechnology), 1:1000; c-Jun (sc-1694; Santa Cruz Biotechnology), 1:1000; Bax (GTX109683; GeneTex), 1:1000; Cytochrome *c* (11940; Cell Signaling Technology), 1:1000; Caspase-9 (GTX112888; GeneTex), 1:1000; Caspase-3 (GTX110543; GeneTex), 1:1000; PARP1 (GTX100573; GeneTex), 1:1000; Akt (GTX121937; GeneTex), 1:1000; BCL-2 (GTX127958; GeneTex), 1:1000; BIRC2 (GTX110087; GeneTex), 1:1000; Catalase (GTX110704; GeneTex), 1:500; Clusterin (GTX101236; GeneTex), 1:800; ERK1/2 (sc-292838; Santa Cruz Biotechnology), 1:1000; GSK3β (GTX111192; GeneTex), 1:1000; HIF1A (GTX127309; GeneTex), 1:800; HMOX1 (GTX101147; GeneTex), 1:800; HSPB1 (GTX101145; GeneTex), 1:1000; mTOR (GTX101557; GeneTex), 1:1000; p21 Cip1 (GTX100444; GeneTex), 1:800; p27 Kip1 (GTX100446; GeneTex), 1:800; STAT5 (sc-835; Santa Cruz Biotechnology), 1:600; STAT6 (GTX113273; GeneTex), 1:1000; TNFRSF1A (GTX102718; GeneTex), 1:1000; TP53 (sc-6243; Santa Cruz Biotechnology), 1:1000; XIAP (GTX113130; GeneTex), 1:200; and β-Actin (A5441; Sigma Aldrich), 1:10000.

### Cell culture

The human bronchial epithelial cell line (BEAS-2B) was purchased from the American Type Culture Collection (ATCC) (Rockville, MD). BEAS-2B cells were isolated from normal human bronchial epithelium obtained from autopsy of a non-cancerous individual. Cells were routinely grown in LHC-9 medium (Gibco, Grand Island, NY) at 37°C in an atmosphere of 5% CO_2_/95% air as recommended by ATCC. LHC-9 is a defined, serum-free medium which is prepared by mixing LHC basal medium with growth factors, cytokines, and supplements; and has been described previously [35].

### Cd treatment

Cells were grown to 75% confluence in 60 mM cell culture dishes and then rinsed with Hank's balanced salt solution (HBSS) before they were either sham-exposed or treated with different concentrations of CdCl_2_ in LHC-9 medium. Cells were pretreated with GSH or p38 MAPK/JNK inhibitors for 1 h before the addition of Cd.

### Cell lysate preparation

After treatment, cells were rinsed with PBS and extracted with Lysis buffer 6 (provided in the Human Phospho-MAPK and Human Phospho-Kinase Array Kit) or Lysis buffer 17 (provided in the Human Apoptosis Array Kit). After incubating and rocking at 4°C for 30 min, the samples were centrifuged at 14,000 g for 5 min, supernates were transferred into clean tubes and subjected to protein quantification. For subcellular proteins preparation, the cytosolic fraction was prepared using the Subcellular Protein Fractionation Kit for Cultured Cells in accordance with the manufacturer's instructions.

### Protein array analysis

Analysis of proteins by Human Phospho-MAPK, Human Phospho-Kinase and Human Apoptosis Arrays was conducted following the manufacturer's instructions. In brief, protein sample was incubated with each array at 4°C overnight on a rocking platform shaker. The unbound proteins were removed, and the arrays were washed three times with washing buffer. Arrays were incubated with the primary antibody solution for 2 h at room temperature and then washed three times with washing buffer. The secondary antibody solution was then added to the arrays on a rocking platform shaker for 1 h. The arrays were washed three times with washing buffer, and protein spots were visualized using the chemiluminescence detection reagents supplied in the Array Kits. The intensity score of each duplicated array spot was measured with the ImageJ (version 1.37v) software program, and the averaged intensity was calculated by subtracting the averaged background signal. The fold change was obtained by comparing Cd-treated samples with the untreated control (indicated as a value of 1). The identity and the respective coordinates of all the antibodies on the arrays can be found in “Supplementary Material”.

### Conditions of western blot

For western blot analysis, equal amounts of proteins were fractionated on appropriate percentage of SDS–polyacrylamide gel and transferred onto polyvinylidene difluoride membranes. After the transfer, the membranes were blocked with 5% nonfat dry milk in PBS containing 0.05% Tween 20 and probed with various primary antibodies. After incubation with secondary antibodies, immunoblots were visualized with the enhanced chemiluminescence detection kit (GE Healthcare). Reproducibility was confirmed in three separate experiments and representative data were shown.

### Fluorescence microscopy

The Mitochondria Apoptosis Detection Kit (JC-1) was purchased from GenScript (Piscataway, NJ). This Kit provides a simple, fluorescent-based method for distinguishing between healthy and apoptotic cells by detecting the loss of the mitochondrial transmembrane potential (Δψ). The kit utilizes the unique lipophilic cationic dye (5, 5′, 6, 6′-tetrachloro-1, 1′, 3, 3′-tetraethylbenzimidazolylcarbocyanine iodine, commonly known as JC-1) for detection. In healthy cells, the negative charge established by the intact mitochondrial membrane potential allows the lipophilic dye, bearing a delocalized positive charge, to enter the mitochondrial matrix where it accumulates. With the increasing of the concentration of JC-1, it aggregates and becomes fluorescent red. When the mitochondrial potential collapses in apoptotic cells, JC-1 just exists as monomers and do not accumulate within the mitochondria. When dispersed in this manner, JC-1 remains in the cytoplasm in a green fluorescent monomeric form. The fluorescent signals were examined by laser-scanning confocal microscopy (OLYMPUS FV1000), using FITC channel for green monomers and PI channel for red aggregates. Reproducibility was confirmed in three separate experiments and representative data were shown.

### Measurement of ROS generation

ROS levels were determined by incubating the cells in LHC-9 media with 2 μM dihydroethidium (DHE, Molecular Probes) for 30 min at 37°C. Cells were washed three times with DPBS and replaced with new LHC-9 media, and then treated with 30 or 100 μM CdCl_2_ for different time. After treatment, cells were trypsinized and fluorescence was measured using BD Accuri™ C6 flow cytometer with excitation and emission wavelengths at 535 nm and 610 nm, respectively.

### Bioinformatic analyses

Differentially-stimulated/inhibited proteins were listed and the data were analyzed through the use of QIAGEN's Ingenuity^®^ Pathway Analysis (IPA^®^, QIAGEN Redwood City, www.qiagen.com/ingenuity), to highlight the relationships among candidate proteins using networks and canonical pathways. Ingenuity Knowledge Base, the core behind IPA^®^, provides a wide range of high-quality detailed information, including direct and indirect protein interaction networks, thus aiding the generation of hypotheses for a comprehensive analysis of a large number of data.

### Data analysis

For antibody array analysis, protein levels with differences of higher than ± 2 folds (i.e. ≥ 2 or ≤ 0.5) are considered as candidates that are more importantly participated in the Cd toxicity pathway.

## SUPPLEMENTARY MATERIALS TABLES AND FIGURES


