# Supplementary material for: Proteome profiling of cadmium-induced apoptosis by antibody array analyses in human bronchial epithelial cells
Source: Oncotarget. 2015 Dec 23;7(5):6146–58. doi: 10.18632/oncotarget.6738 (PMC4868746; doi:10.18632/oncotarget.6738)
Supplement: Supplementary file 1 [file oncotarget-07-6146-s001.pdf]

Proteome profiling of cadmium-induced apoptosis by antibody array analyses in human bronchial epithelial cells

Supplementary Materials

Supplementary Table S1: Human phospho-MAPK array coordinates

Coordinate	Target/Control	Alternate Nomenclature	Phosphorylation Site Detected
A1, A2	Reference Spots	–	–
A21, A22	Reference Spots	–	–
B3, B4	Akt1	PKBα, RACα	S473
B5, B6	Akt2	PKBβ, RACβ	S474
B7, B8	Akt3	PKBγ, RACγ	S472
B9, B10	Akt pan	–	S473, S474, S472
B11, B12	CREB	–	S133
B13, B14	ERK1	MAPK3, p44 MAPK	T202/Y204
B15, B16	ERK2	MAPK1, p42 MAPK	T185/Y187
B17, B18	GSK-3α/β	GSK3A/GSK3B	S21/S9
B19, B20	GSK-3β	GSK3B	S9
C3, C4	HSP27	HSPB1, SRP27	S78/S82
C5, C6	JNK1	MAPK8, SAPK1γ	T183/Y185
C7, C8	JNK2	MAPK9, SAPK1α	T183/Y185
C9, C10	JNK3	MAPK10, SAPK1β	T221/Y223
C11, C12	JNK pan	–	T183/Y185, T221/Y223
C13, C14	MKK3	MEK3, MAP2K3	S218/T222
C15, C16	MKK6	MEK6, MAP2K6	S207/T211
C17, C18	MSK2	RSKβ, RPS6KA4	S360
D3, D4	p38α	MAPK14, SAPK2A, CSBP1	T180/Y182
D5, D6	p38β	MAPK11, SAPK2B, p38-2	T180/Y182
D7, D8	p38δ	MAPK13, SAPK4	T180/Y182
D9, D10	p38γ	MAPK12, SAPK3, ERK6	T183/Y185
D11, D12	p53	–	S46
D13, D14	p70 S6 Kinase	S6K1, p70α, RPS6KB1	T421/S424
D15, D16	RSK1	MAPKAPK1α, RPS6KA1	S380
D17, D18	RSK2	ISPK-1, RPS6KA3	S386
D19, D20	TOR	–	S2448
E19, E20	PBS	Control (–)	–
F1, F2	Reference Spots	–	–

Human Phospho-MAPK Array Coordinates

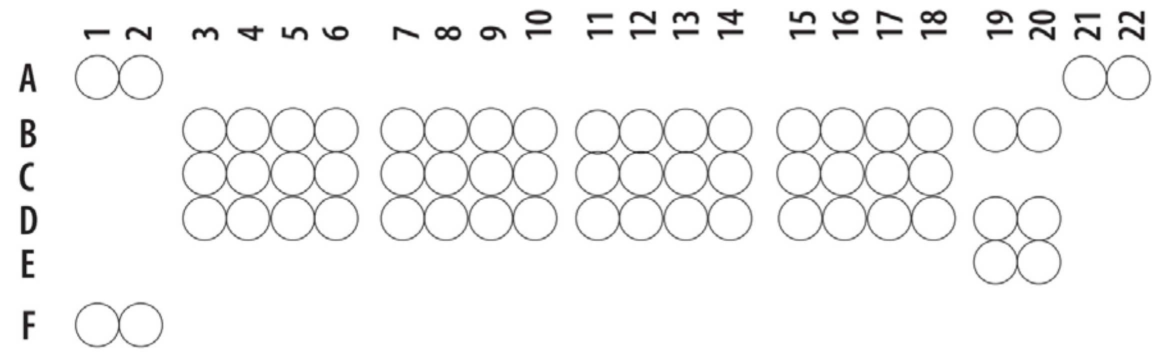

Supplementary Table S2: Human phospho-kinase array coordinates

Coordinate	Target/Control	Phosphorylation Site
A1, A2	Reference Spot	–
A3, A4	p38α	T180/Y182
A5, A6	ERK1/2	T202/Y204, T185/Y187
A7, A8	JNK pan	T183/Y185, T221/Y223
A9, A10	GSK-3α/β	S21/S9
A13, A14	p53	S392
A17, A18	Reference Spot	–
B3, B4	EGF R	Y1086
B5, B6	MSK1/2	S376/S360
B7, B8	AMPKα1	T174
B9, B10	Akt	S473
B11, B12	Akt	T308
B13, B14	p53	S46
C1, C2	TOR	S2448
C3, C4	CREB	S133
C5, C6	HSP27	S78/S82
C7, C8	AMPKα2	T172
C9, C10	β-Catenin	–
C11, C12	p70 S6 Kinase	T389
C13, C14	p53	S15
C15, C16	c-Jun	S63
D1, D2	Src	Y419
D3, D4	Lyn	Y397
D5, D6	Lck	Y394
D7, D8	STAT2	Y689
D9, D10	STAT5a	Y694

D11, D12	p70 S6 Kinase	T421/S424
D13, D14	RSK1/2/3	S380/S386/S377
D15, D16	eNOS	S1177
E1, E2	Fyn	Y420
E3, E4	Yes	Y426
E5, E6	Fgr	Y412
E7, E8	STAT6	Y641
E9, E10	STAT5b	Y699
E11, E12	STAT3	Y705
E13, E14	p27	T198
E15, E16	PLC-γ1	Y783
F1, F2	Hck	Y411
F3, F4	Chk-2	T68
F5, F6	FAK	Y397
F7, F8	PDGF Rβ	Y751
F9, F10	STAT5a/b	Y694/Y699
F11, F12	STAT3	S727
F13, F14	WNK1	T60
F15, F16	PYK2	Y402
G1, G2	Reference Spot	–
G3, G4	PRAS40	T246
G9, G10	PBS (Negative Control)	–
G11, G12	HSP60	–
G17, G18	PBS (Negative Control)	–

Human Phospho-Kinase Array Coordinates

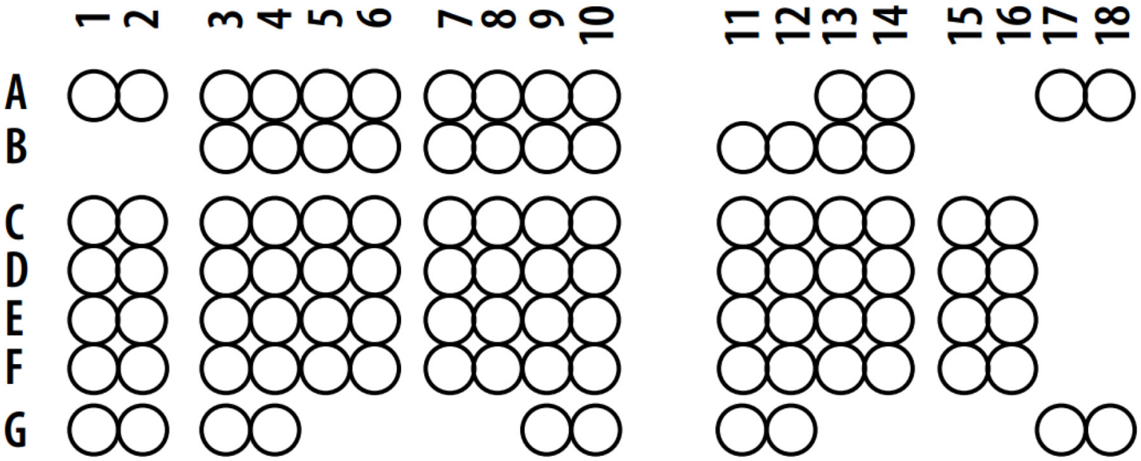

Supplementary Table S3: Human apoptosis array coordinates

Coordinate	Target/Control	Coordinate	Target/Control
A1, A2	Reference Spots	C13, C14	HO-2/HMOX2
A23, A24	Reference Spots	C15, C16	HSP27
B1, B2	Bad	C17, C18	HSP60
B3, B4	Bax	C19, C20	HSP70
B5, B6	Bcl-2	C21, C22	HTRA2/Omi
B7, B8	Bcl-x	C23, C24	Livin
B9, B10	Pro-caspase-3	D1, D2	PON2
B11, B12	Cleaved caspase-3	D3, D4	p21/CIP1/CDKN1A
B13, B14	Catalase	D5, D6	p27/Kip1
B15, B16	cIAP-1	D7, D8	Phospho-p53 (S15)
B17, B18	cIAP-2	D9, D10	Phospho-p53 (S46)
B19, B20	Claspin	D11, D12	Phospho-p53 (S392)
B21, B22	Clusterin	D13, D14	Phospho-Rad17 (S635)
B23, B24	Cytochrome c	D15, D16	SMAC/Diablo
C1, C2	TRAIL R1/DR4	D17, D18	Survivin
C3, C4	TRAIL R2/DR5	D19, D20	TNF-RI/TNFRSF1A
C5, C6	FADD	D21, D22	XIAP
C7, C8	Fas/TNFRSF6/CD95	D23, D24	PBS (Negative Control)
C9, C10	HIF-1α	E1, E2	Reference Spots
C11, C12	HO-1/HMOX1/HSP32		

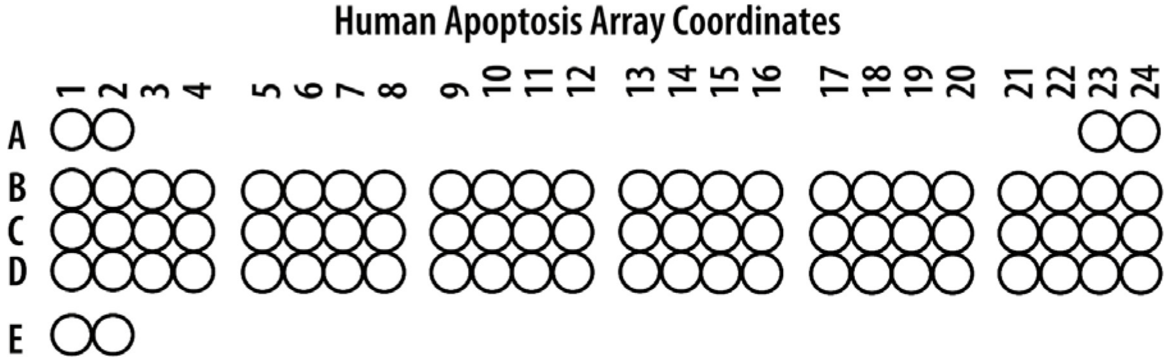

Supplementary Table S4: Human phospho-MAPK array experimental data of BEAS-2B cells treated with 100 μM $CdCl_2$ for 3 h

Target		Mean of spot duplicates (Set 1)	Fold	Mean of spot duplicates (Set 2)	Fold	Mean fold of set 1 & 2
Akt1 (S473)	Control	501.124	1.000	499.093	1.000	1.000
	Cd	79.133	0.158	93.014	0.186	0.172
Akt2 (S474)	Control	383.534	1.000	375.202	1.000	1.000
	Cd	383.063	0.999	369.926	0.986	0.992
Akt3 (S472)	Control	21.935	1.000	18.663	1.000	1.000
	Cd	21.993	1.003	23.304	1.249	1.116
Akt pan (S473, S474, S472)	Control	150.510	1.000	158.546	1.000	1.000
	Cd	55.190	0.367	51.611	0.326	0.346
CREB (S133)	Control	50.037	1.000	48.013	1.000	1.000
	Cd	126.980	2.538	122.363	2.549	2.543
ERK1 (T202/Y204)	Control	524.116	1.000	509.179	1.000	1.000
	Cd	20.183	0.039	24.246	0.048	0.043
ERK2 (T185/Y187)	Control	1081.539	1.000	1087.712	1.000	1.000
	Cd	252.719	0.234	252.069	0.232	0.233
GSK-3α/β (S21/S9)	Control	490.262	1.000	473.967	1.000	1.000
	Cd	437.765	0.893	419.268	0.885	0.889
GSK-3β (S9)	Control	874.017	1.000	899.010	1.000	1.000
	Cd	631.969	0.723	640.381	0.712	0.718
HSP27 (S78/S82)	Control	399.391	1.000	404.736	1.000	1.000
	Cd	784.707	1.965	806.331	1.992	1.979
JNK1 (T183/Y185)	Control	9.761	1.000	8.623	1.000	1.000
	Cd	102.653	10.517	98.284	11.398	10.930
JNK2 (T183/Y185)	Control	58.894	1.000	62.110	1.000	1.000
	Cd	498.779	8.469	506.917	8.162	8.311
JNK3 (T221/Y223)	Control	21.909	1.000	19.732	1.000	1.000
	Cd	16.259	0.742	17.443	0.884	0.809
JNK pan (T183/Y185, T221/Y223)	Control	142.913	1.000	165.899	1.000	1.000
	Cd	781.023	5.465	759.829	4.580	4.990
MKK3 (S218/T222)	Control	75.770	1.000	70.428	1.000	1.000
	Cd	62.113	0.820	64.941	0.922	0.869
MKK6 (S207/T211)	Control	56.308	1.000	57.566	1.000	1.000
	Cd	43.267	0.768	47.353	0.823	0.796
MSK2 (S360)	Control	119.978	1.000	133.442	1.000	1.000
	Cd	189.105	1.576	173.588	1.301	1.431
p38α (T180/Y182)	Control	11.476	1.000	11.631	1.000	1.000
	Cd	287.529	25.055	297.985	25.620	25.339
p38β (T180/Y182)	Control	57.859	1.000	64.342	1.000	1.000
	Cd	55.609	0.961	50.524	0.785	0.869

p38δ (T180/Y182)	Control	367.063	1.000	384.638	1.000	1.000
	Cd	1137.590	3.099	1136.525	2.955	3.025
p38γ (T183/Y185)	Control	96.256	1.000	105.057	1.000	1.000
	Cd	235.825	2.450	232.855	2.216	2.328
p53 (S46)	Control	2198.592	1.000	2253.575	1.000	1.000
	Cd	1878.436	0.854	1891.655	0.839	0.847
p70 S6 Kinase (T421/S424)	Control	5.747	1.000	5.100	1.000	1.000
	Cd	6.949	1.209	7.991	1.567	1.377
RSK1 (S380)	Control	121.066	1.000	125.575	1.000	1.000
	Cd	113.978	0.941	105.432	0.840	0.890
RSK2 (S386)	Control	50.274	1.000	52.433	1.000	1.000
	Cd	30.280	0.602	32.262	0.615	0.609
TOR (S2448)	Control	207.262	1.000	191.382	1.000	1.000
	Cd	51.191	0.247	40.861	0.214	0.231

Data are detailed spot densities and expressed as mean values from 2 independent experiments. The fold change was obtained by comparing Cd-treated samples with the untreated control (indicated as a value of 1).

Supplementary Table S5: Human phospho-kinase array experimental data of BEAS-2B cells treated with 30 μM $CdCl_2$ for 6, 12, and 24 h

Target		Mean of spot duplicates (Set 1)	Fold	Mean of spot duplicates (Set 2)	Fold	Mean fold of set 1 & 2
p38α (T180/Y182)	0 h	245.640	1.000	222.589	1.000	1.000
	6 h	665.736	2.710	660.082	2.965	2.832
	12 h	819.190	3.335	795.967	3.576	3.450
	24 h	723.360	2.945	712.392	3.200	3.066
ERK1/2 (T202/Y204, T185/Y187)	0 h	679.698	1.000	671.773	1.000	1.000
	6 h	787.432	1.159	760.818	1.133	1.146
	12 h	752.127	1.107	767.412	1.142	1.124
	24 h	528.571	0.778	504.920	0.752	0.765
JNK pan (T183/Y185, T221/Y223)	0 h	155.654	1.000	154.040	1.000	1.000
	6 h	324.939	2.088	333.297	2.164	2.125
	12 h	413.904	2.659	425.562	2.763	2.711
	24 h	437.266	2.809	444.502	2.886	2.847
GSK-3α/β (S21/S9)	0 h	492.388	1.000	488.206	1.000	1.000
	6 h	565.730	1.149	514.543	1.054	1.102
	12 h	411.758	0.836	421.914	0.864	0.850
	24 h	402.134	0.817	400.031	0.819	0.818
p53 (S392)	0 h	1696.102	1.000	1745.138	1.000	1.000
	6 h	1622.268	0.956	1634.306	0.936	0.946
	12 h	1623.780	0.957	1612.291	0.924	0.940
	24 h	1346.684	0.794	1345.498	0.771	0.782

EGF R (Y1086)	0 h	390.279	1.000	385.811	1.000	1.000
	6 h	559.480	1.434	533.493	1.383	1.408
	12 h	306.156	0.784	295.567	0.766	0.775
	24 h	118.116	0.303	114.662	0.297	0.300
MSK1/2 (S376/S360)	0 h	199.645	1.000	182.792	1.000	1.000
	6 h	265.963	1.332	281.624	1.541	1.432
	12 h	254.782	1.276	258.625	1.415	1.342
	24 h	212.264	1.063	208.816	1.142	1.101
AMPKα1 (T174)	0 h	200.496	1.000	191.665	1.000	1.000
	6 h	259.502	1.294	269.992	1.409	1.350
	12 h	123.259	0.615	131.958	0.688	0.651
	24 h	193.514	0.965	196.127	1.023	0.994
Akt (S473)	0 h	960.303	1.000	855.307	1.000	1.000
	6 h	1024.307	1.067	1020.366	1.193	1.126
	12 h	1087.490	1.132	1085.240	1.269	1.197
	24 h	572.120	0.596	576.221	0.674	0.632
Akt (T308)	0 h	221.483	1.000	240.710	1.000	1.000
	6 h	304.780	1.376	317.266	1.318	1.346
	12 h	268.201	1.211	292.382	1.215	1.213
	24 h	202.085	0.912	192.349	0.799	0.853
p53 (S46)	0 h	1268.249	1.000	1322.966	1.000	1.000
	6 h	1236.464	0.975	1249.800	0.945	0.959
	12 h	1198.607	0.945	1198.614	0.906	0.925
	24 h	969.898	0.765	969.257	0.733	0.748
TOR (S2448)	0 h	170.604	1.000	161.173	1.000	1.000
	6 h	181.003	1.061	176.036	1.092	1.076
	12 h	168.813	0.990	167.736	1.041	1.014
	24 h	120.110	0.704	120.197	0.746	0.724
CREB (S133)	0 h	382.525	1.000	369.023	1.000	1.000
	6 h	503.405	1.316	481.816	1.306	1.311
	12 h	660.428	1.726	627.114	1.699	1.713
	24 h	671.161	1.755	653.974	1.772	1.763
HSP27 (S78/S82)	0 h	114.408	1.000	97.573	1.000	1.000
	6 h	168.670	1.474	155.297	1.592	1.528
	12 h	183.491	1.604	184.920	1.895	1.738
	24 h	439.308	3.840	437.313	4.482	4.135
AMPKα2 (T172)	0 h	227.157	1.000	219.384	1.000	1.000
	6 h	388.362	1.710	403.542	1.839	1.773
	12 h	351.119	1.546	340.856	1.554	1.550
	24 h	352.636	1.552	362.435	1.652	1.601

β-Catenin	0 h	363.008	1.000	422.602	1.000	1.000
	6 h	722.489	1.990	714.854	1.692	1.830
	12 h	621.775	1.713	642.054	1.519	1.609
	24 h	441.531	1.216	416.846	0.986	1.093
p70S6 Kinase (T389)	0 h	59.255	1.000	65.744	1.000	1.000
	6 h	85.936	1.450	83.043	1.263	1.352
	12 h	43.499	0.734	42.397	0.645	0.687
	24 h	49.514	0.836	48.399	0.736	0.783
p53 (S15)	0 h	1128.811	1.000	1170.816	1.000	1.000
	6 h	1147.771	1.017	1128.620	0.964	0.990
	12 h	802.030	0.711	841.798	0.719	0.715
	24 h	737.689	0.654	693.264	0.592	0.622
c-Jun (S63)	0 h	214.982	1.000	228.699	1.000	1.000
	6 h	1027.241	4.778	1013.019	4.429	4.598
	12 h	1299.765	6.046	1362.902	5.959	6.001
	24 h	1197.815	5.572	1228.344	5.371	5.468
Src (Y419)	0 h	162.438	1.000	150.212	1.000	1.000
	6 h	162.105	0.998	163.538	1.089	1.042
	12 h	145.053	0.893	144.853	0.964	0.927
	24 h	114.595	0.705	114.609	0.763	0.733
Lyn (Y397)	0 h	103.952	1.000	97.899	1.000	1.000
	6 h	92.979	0.894	87.952	0.898	0.896
	12 h	106.810	1.027	101.346	1.035	1.031
	24 h	129.178	1.243	125.329	1.280	1.261
Lck (Y394)	0 h	56.689	1.000	52.599	1.000	1.000
	6 h	41.031	0.724	42.142	0.801	0.761
	12 h	46.122	0.814	48.212	0.917	0.863
	24 h	71.773	1.266	66.183	1.258	1.262
STAT2 (Y689)	0 h	373.822	1.000	386.126	1.000	1.000
	6 h	504.010	1.348	513.155	1.329	1.338
	12 h	466.010	1.247	471.160	1.220	1.233
	24 h	466.010	1.247	467.439	1.211	1.228
STAT5a (Y694)	0 h	228.171	1.000	218.578	1.000	1.000
	6 h	295.025	1.293	283.295	1.296	1.293
	12 h	288.180	1.263	276.594	1.265	1.263
	24 h	301.408	1.321	327.535	1.498	1.406
p70S6 Kinase (T421/S424)	0 h	189.728	1.000	190.402	1.000	1.000
	6 h	259.689	1.369	264.577	1.390	1.379
	12 h	180.106	0.949	182.451	0.958	0.954
	24 h	167.857	0.885	167.092	0.878	0.881

RSK1/2/3 (S380/S386/S377)	0 h	293.290	1.000	298.408	1.000	1.000
	6 h	282.084	0.962	281.766	0.944	0.953
	12 h	242.567	0.827	239.093	0.801	0.814
	24 h	397.759	1.356	393.939	1.320	1.338
eNOS (S1177)	0 h	109.954	1.000	113.510	1.000	1.000
	6 h	139.574	1.269	152.817	1.346	1.308
	12 h	130.646	1.188	141.155	1.244	1.216
	24 h	125.083	1.138	124.679	1.098	1.118
Fyn (Y420)	0 h	141.792	1.000	141.800	1.000	1.000
	6 h	123.703	0.872	128.138	0.904	0.888
	12 h	128.367	0.905	130.797	0.922	0.914
	24 h	102.064	0.720	100.214	0.707	0.713
Yes (Y426)	0 h	204.052	1.000	189.457	1.000	1.000
	6 h	197.299	0.967	191.132	1.009	0.987
	12 h	191.728	0.940	178.310	0.941	0.940
	24 h	164.533	0.806	162.604	0.858	0.831
Fgr (Y412)	0 h	61.842	1.000	46.736	1.000	1.000
	6 h	53.883	0.871	43.170	0.924	0.894
	12 h	53.045	0.858	46.328	0.991	0.915
	24 h	60.999	0.986	54.630	1.169	1.065
STAT6 (Y641)	0 h	110.397	1.000	102.387	1.000	1.000
	6 h	193.691	1.754	246.962	2.412	2.071
	12 h	233.859	2.118	249.123	2.433	2.270
	24 h	213.348	1.933	215.718	2.107	2.016
STAT5b (Y699)	0 h	45.589	1.000	54.579	1.000	1.000
	6 h	172.256	3.778	183.196	3.357	3.549
	12 h	201.899	4.429	208.690	3.824	4.099
	24 h	144.802	3.176	139.900	2.563	2.842
STAT3 (Y705)	0 h	101.393	1.000	96.248	1.000	1.000
	6 h	176.647	1.742	180.128	1.871	1.805
	12 h	95.802	0.945	90.280	0.938	0.942
	24 h	105.313	1.039	107.053	1.112	1.075
p27 (T198)	0 h	28.069	1.000	30.168	1.000	1.000
	6 h	33.589	1.197	33.382	1.107	1.150
	12 h	25.119	0.895	24.703	0.819	0.856
	24 h	28.121	1.002	30.341	1.006	1.004
PLC-γ1 (Y783)	0 h	124.755	1.000	124.418	1.000	1.000
	6 h	153.472	1.230	186.841	1.502	1.366
	12 h	171.311	1.373	168.096	1.351	1.362
	24 h	153.257	1.228	141.631	1.138	1.183

Hck (Y411)	0 h	144.685	1.000	153.988	1.000	1.000
	6 h	160.041	1.106	161.315	1.048	1.078
	12 h	152.598	1.055	154.753	1.005	1.029
	24 h	114.853	0.794	104.771	0.680	0.735
Chk-2 (T68)	0 h	285.327	1.000	268.784	1.000	1.000
	6 h	326.346	1.144	317.631	1.182	1.162
	12 h	295.611	1.036	288.143	1.072	1.053
	24 h	217.787	0.763	213.066	0.793	0.778
FAK (Y397)	0 h	187.013	1.000	163.808	1.000	1.000
	6 h	209.884	1.122	163.444	0.998	1.064
	12 h	194.958	1.042	170.366	1.040	1.041
	24 h	126.833	0.678	122.853	0.750	0.712
PDGF Rβ (Y751)	0 h	63.156	1.000	55.463	1.000	1.000
	6 h	78.498	1.243	77.896	1.404	1.318
	12 h	72.142	1.142	77.248	1.393	1.259
	24 h	69.021	1.093	66.276	1.195	1.141
STAT5a/b (Y694/Y699)	0 h	161.591	1.000	136.507	1.000	1.000
	6 h	223.709	1.384	241.850	1.772	1.562
	12 h	274.657	1.700	303.715	2.225	1.940
	24 h	270.313	1.673	246.703	1.807	1.734
STAT3 (S727)	0 h	49.034	1.000	55.617	1.000	1.000
	6 h	124.132	2.532	173.793	3.125	2.847
	12 h	63.763	1.300	65.714	1.182	1.237
	24 h	63.918	1.304	76.292	1.372	1.340
WNK1 (T60)	0 h	1045.384	1.000	1140.931	1.000	1.000
	6 h	1549.620	1.482	1514.198	1.327	1.401
	12 h	1166.632	1.116	1036.972	0.909	1.008
	24 h	1057.450	1.012	1041.994	0.913	0.960
PYK2 (Y402)	0 h	87.538	1.000	85.262	1.000	1.000
	6 h	128.462	1.468	129.804	1.522	1.495
	12 h	98.706	1.128	110.149	1.292	1.209
	24 h	84.060	0.960	80.332	0.942	0.951
PRAS40 (T246)	0 h	785.907	1.000	783.596	1.000	1.000
	6 h	768.492	0.978	748.362	0.955	0.966
	12 h	744.068	0.947	723.522	0.923	0.935
	24 h	567.480	0.722	565.132	0.721	0.722
HSP60	0 h	324.045	1.000	288.985	1.000	1.000
	6 h	605.500	1.869	606.944	2.100	1.978
	12 h	484.679	1.496	487.185	1.686	1.585
	24 h	410.733	1.268	406.653	1.407	1.333

Data are detailed spot densities and expressed as mean values from 2 independent experiments. The fold change was obtained by comparing Cd-treated samples with the untreated control (indicated as a value of 1).

Supplementary Table S6: Human apoptosis array experimental data of BEAS-2B cells treated with 30 μM $CdCl_2$ for 24 h

Target		Mean of spot duplicates (Set 1)	Fold	Mean of spot duplicates (Set 2)	Fold	Mean fold of set 1 & 2
Bad	Control	339.072	1.000	307.284	1.000	1.000
	Cd	339.587	1.002	313.459	1.020	1.010
Bax	Control	213.560	1.000	192.088	1.000	1.000
	Cd	381.014	1.784	370.298	1.928	1.852
Bcl-2	Control	103.963	1.000	117.888	1.000	1.000
	Cd	101.536	0.977	106.826	0.906	0.939
Bcl-x	Control	41.659	1.000	72.977	1.000	1.000
	Cd	44.697	1.073	73.469	1.007	1.031
Pro-caspase-3	Control	1217.254	1.000	1205.082	1.000	1.000
	Cd	1026.746	0.843	1011.958	0.840	0.842
Cleaved caspase-3	Control	261.067	1.000	150.148	1.000	1.000
	Cd	518.712	1.987	490.232	3.265	2.454
Catalase	Control	122.528	1.000	129.589	1.000	1.000
	Cd	201.634	1.646	205.796	1.588	1.616
cIAP-1	Control	306.833	1.000	309.813	1.000	1.000
	Cd	36.004	0.117	38.974	0.126	0.122
cIAP-2	Control	94.807	1.000	124.906	1.000	1.000
	Cd	79.358	0.837	79.900	0.640	0.725
Claspin	Control	312.843	1.000	331.120	1.000	1.000
	Cd	173.614	0.555	175.542	0.530	0.542
Clusterin	Control	125.558	1.000	119.500	1.000	1.000
	Cd	259.574	2.067	258.528	2.163	2.114
Cytochrome c	Control	226.787	1.000	205.236	1.000	1.000
	Cd	228.640	1.008	225.668	1.100	1.052
TRAIL R1/DR4	Control	486.885	1.000	495.589	1.000	1.000
	Cd	501.280	1.030	501.750	1.012	1.021
TRAIL R2/DR5	Control	495.777	1.000	486.871	1.000	1.000
	Cd	530.460	1.070	521.224	1.071	1.070
FADD	Control	352.667	1.000	353.443	1.000	1.000
	Cd	371.243	1.053	374.479	1.060	1.056
Fas/TNFRSF6/CD95	Control	619.060	1.000	606.731	1.000	1.000
	Cd	631.248	1.020	624.036	1.029	1.024
HIF-1α	Control	358.130	1.000	381.667	1.000	1.000
	Cd	851.632	2.378	860.036	2.253	2.314
HO-1/HMOX1/HSP32	Control	310.154	1.000	244.051	1.000	1.000
	Cd	672.055	2.167	646.177	2.648	2.379

HO-2/HMOX2	Control	395.649	1.000	380.104	1.000	1.000
	Cd	409.776	1.036	400.478	1.054	1.044
HSP27	Control	436.717	1.000	432.289	1.000	1.000
	Cd	924.061	2.116	922.205	2.133	2.125
HSP60	Control	427.526	1.000	431.357	1.000	1.000
	Cd	530.906	1.242	537.346	1.246	1.244
HSP70	Control	420.807	1.000	417.311	1.000	1.000
	Cd	460.525	1.094	438.273	1.050	1.072
HTRA2/Omi	Control	326.893	1.000	335.883	1.000	1.000
	Cd	221.905	0.679	231.831	0.690	0.685
Livin	Control	33.716	1.000	28.338	1.000	1.000
	Cd	32.728	0.971	31.890	1.125	1.041
PON2	Control	93.788	1.000	78.956	1.000	1.000
	Cd	87.426	0.932	86.588	1.097	1.007
p21/CIP1/CDKN1A	Control	136.791	1.000	167.210	1.000	1.000
	Cd	65.091	0.476	75.003	0.449	0.461
p27/Kip1	Control	95.858	1.000	131.020	1.000	1.000
	Cd	61.417	0.641	79.021	0.603	0.619
Phospho-p53 (S15)	Control	896.225	1.000	907.614	1.000	1.000
	Cd	553.582	0.618	557.854	0.615	0.616
Phospho-p53 (S46)	Control	636.940	1.000	653.143	1.000	1.000
	Cd	376.267	0.591	376.767	0.577	0.584
Phospho-p53 (S392)	Control	921.433	1.000	921.414	1.000	1.000
	Cd	723.151	0.785	719.831	0.781	0.783
Phospho-Rad17 (S635)	Control	266.783	1.000	245.316	1.000	1.000
	Cd	264.050	0.990	222.432	0.907	0.950
SMAC/Diablo	Control	579.505	1.000	558.304	1.000	1.000
	Cd	546.440	0.943	530.630	0.950	0.947
Survivin	Control	289.538	1.000	283.179	1.000	1.000
	Cd	148.262	0.512	143.522	0.507	0.509
TNF-RI/TNFRSF1A	Control	27.424	1.000	30.758	1.000	1.000
	Cd	11.494	0.419	16.142	0.525	0.475
XIAP	Control	313.113	1.000	325.148	1.000	1.000
	Cd	25.244	0.081	25.306	0.078	0.079

Data are detailed spot densities and expressed as mean values from 2 independent experiments. The fold change was obtained by comparing Cd-treated samples with the untreated control (indicated as a value of 1).

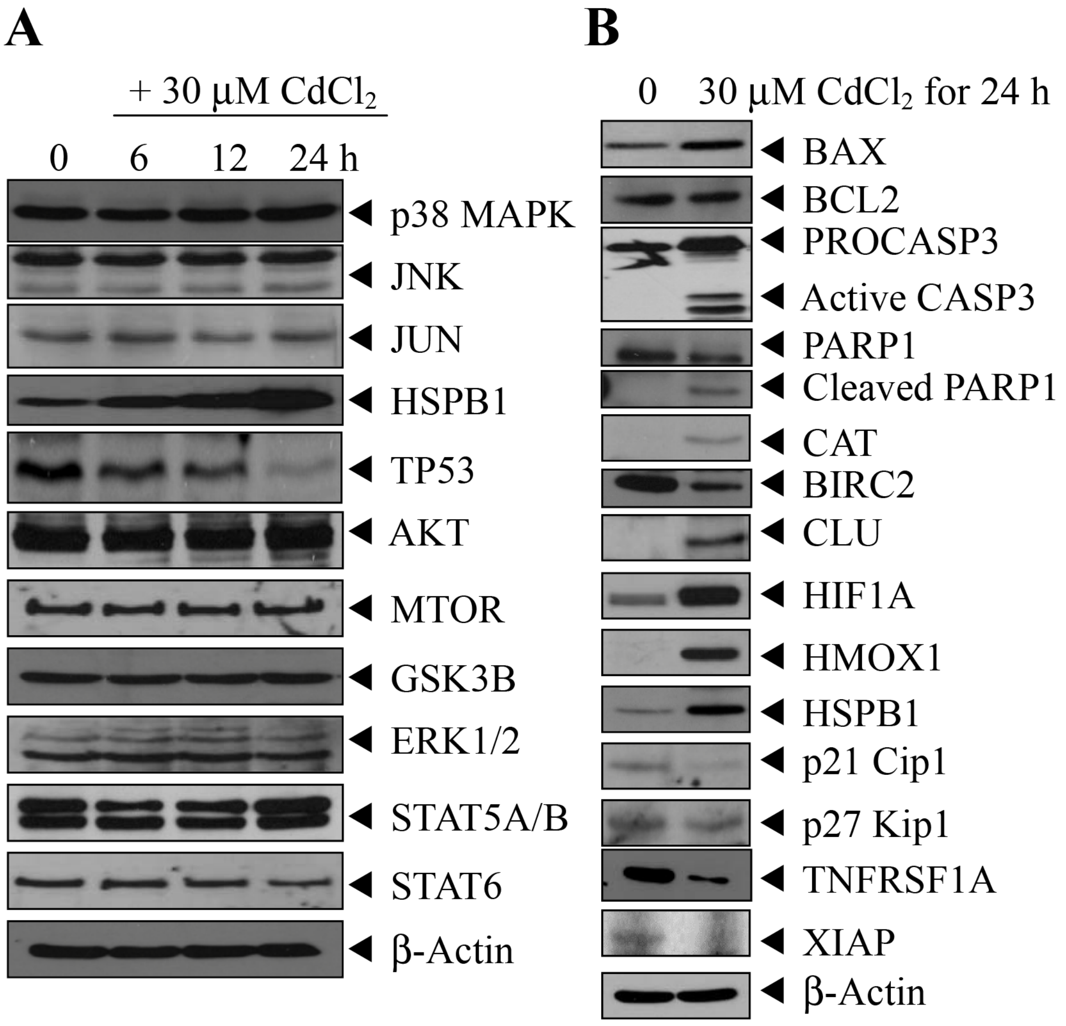

Supplementary Figure S1: Confirmation of protein array data by western blot analyses. BEAS-2B cells were sham-exposed or treated with (**A**) 30 μM $CdCl_2$ for the indicated time or (**B**) 30 μM $CdCl_2$ for 24 h; cells were lysed; and protein extracts were subjected to western blot analysis using various antibodies. The same blot was stripped and reprobed with a monoclonal β-actin antibody to monitor the loading difference. Data are representative of three independent experiments.

A

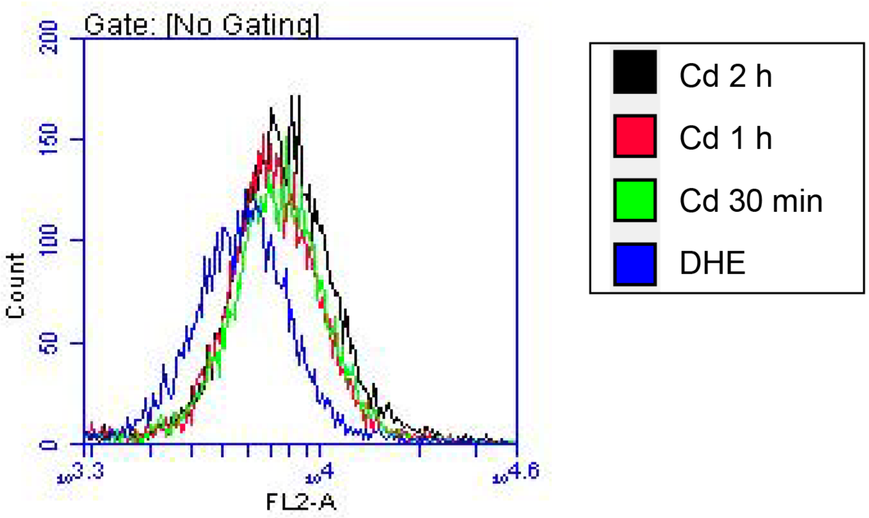

B

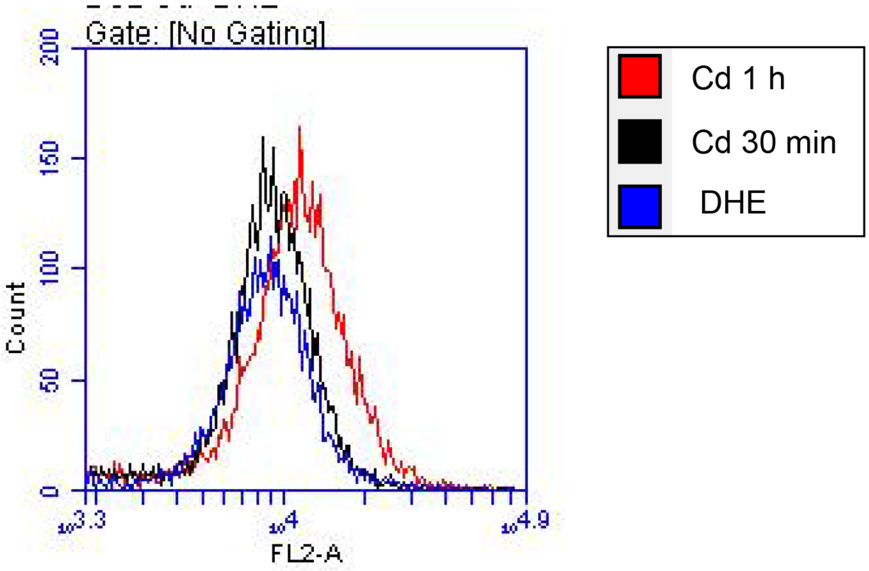

Supplementary Figure S2: Measurement of ROS generation. ROS level of different time points in $CdCl_2$ treated BEAS-2B cells stained with DHE. ROS level increased after treatment with (**A**) 100 µM or (**B**) 30 µM $CdCl_2$. Data are representative of three independent experiments.
